# A higher throughput assay for quantification of melphalan-induced DNA damage in peripheral blood mononuclear cells

**DOI:** 10.1038/s41598-019-55161-3

**Published:** 2019-12-11

**Authors:** Maia van Kan, Kathryn E. Burns, Peter Browett, Nuala A. Helsby

**Affiliations:** Department of Molecular Medicine and Pathology, University of Auckland, Auckland, New Zealand

**Keywords:** Predictive markers, DNA adducts, PCR-based techniques, Myeloma

## Abstract

Inter-individual differences in DNA adduct formation and repair influence the response to melphalan treatment, however, further clinical investigation of this variability requires a logistically feasible and reproducible bioassay. Our improved fluorescence-based QPCR-block assay is robust, has good precision, and improved throughput. It also incorporates direct PCR amplification from melphalan exposed PBMC using commercially available blood tubes and extraction kits to maximise the utility of this assay for future clinical studies. Using this assay we have demonstrated reproducible inter-individual differences in melphalan-induced QPCR-block across individual PBMC donors. As proof-of-principle we assessed nine healthy donors and found a 7.8 fold range in sensitivity following exposure of PBMC *ex vivo*. This likely reflects differences in melphalan transport into cells as well as differences in DNA adduct repair proficiency. This improved bioassay may be useful for assessment of these processes in patients about to receive melphalan treatment.

## Introduction

The myeloablation agent melphalan^1^ is a bi-functional alkylating agent that forms covalent DNA interstrand crosslinks (ICL), preventing both transcription and replication. The number of ICL formed correlates with both *in vitro* cytotoxicity^2^ and patient response^3,4^. Less than 30% of patients achieve complete response^2,5,6^, which may be due to inter-individual differences in the formation or repair of melphalan-induced ICL. Multiple repair complexes, involving more than 130 gene products, facilitate the removal of DNA adducts^7^. As such, identification of genetic polymorphisms that predict sensitivity to melphalan treatment is challenging. A functional assay of adduct formation could provide a useful biomarker to both assess DNA adduct formation and repair proficiency, and to predict response to melphalan treatment.

**Figure 1:**
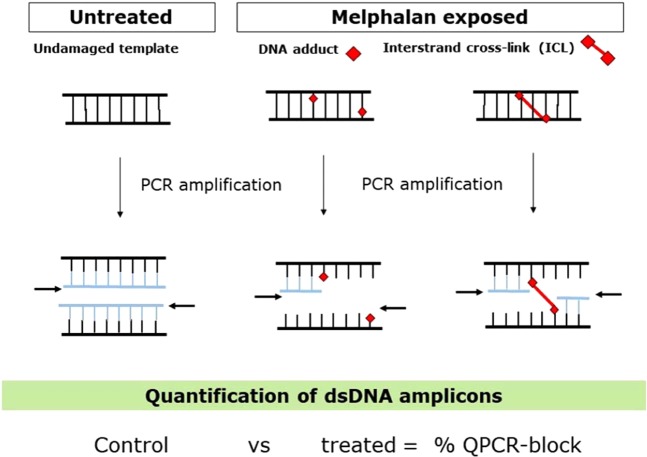
Quantitative PCR-block (QPCR-block) is a well-validated sequence-based approach based on the ability of DNA lesions to block DNA polymerase and stall amplification. Increasing numbers of bulky DNA adducts and interstrand crosslinks (ICL), formed by alkylating agents such as melphalan, in the DNA template proportionally decrease the amplification of a target sequence. Lower amplification reflects higher DNA damage. The amount of target double stranded DNA (dsDNA) amplicon can be then quantified and inhibition of PCR relative to untreated control template is reported as % QPCR-block.

Conventional methods for detection of DNA adduct-induced damage at a global genome level use approaches such as single–cell gel electrophoresis (COMET) assays, detection of DNA fragmentation using terminal deoxynucleotidyl transferase dUTP nick end labelling (TUNEL) assays, immunostaining for DNA damage response proteins at sites of damage (such as γH2AX) or LCMS/MS. However, DNA adducts can also be measured at sequence-specific loci of the genome, using Southern blotting based approaches. Many nucleobase modifications (e.g. UV-induced pyrimidine dimers, strand breaks, bulky adducts and ICL) can form lesions which, if not repaired, interfere with the ability of DNA polymerase to synthesise DNA. Quantitative PCR-block (QPCR-block) is a well-validated approach based on the ability of DNA lesions, such as ICL, to block DNA polymerase and stall amplification. Increasing numbers of adducts in the DNA template proportionally decrease the amplification of a target sequence (Fig. 1). QPCR-block assays have been used to measure the effect of environmental mutagens and UV damage on DNA adduct formation and repair efficiency^8–10^. Moreover, QPCR-block based assays are sequence specific and can be used to determine the extent of DNA adducts formed at genomic loci known to be more susceptible to damage, such as actively transcribed genes and regions of open chromatin. Importantly, melphalan preferentially forms adducts at N-_7_ of guanine and N-_3_ of adenine, with the ICL formed at the following sequences: GnC and AnC^11^.

Recently a long-range QPCR-block assay to detect melphalan-induced DNA adduct formation in peripheral blood mononuclear cells (PBMC) exposed *ex vivo* has been reported^5^. Assessment of patients prior to therapy using this QPCR-block assay could selectively detect responders from non-responders (specificity of 86%, positive predictive value of 92.9%). We now report comparison of the published method with an improved, higher throughput assay and demonstrate the ability of this assay to detect inter-individual differences in DNA adduct formation *ex vivo* in PBMC from healthy donors.

## Results

Initial experiments were undertaken on purified, untreated genomic DNA (gDNA) pooled from six volunteers. While attempting to replicate the published assay^5^, it was found that the polymerase used to amplify the 6.8 kb target sequence in that work^5^ was no longer commercially available. Four alternatives were investigated (Supplementary Fig. 1). LA *Taq* DNA Polymerase (TaKaRa, Japan), Ex *Taq* Hot Start Version Kit (TaKaRa, Japan), and SequalPrep Long PCR Kit (Life Technologies, USA) failed to generate major products specific for the 6.8 kb target amplicon. This may be due to predicted G-quadruplex structures adjacent to the annealing site of the sense primer^12,13^; G-quadruplexes can affect the proof-reading fidelity of long-range PCR DNA polymerases^14^. However, the Phusion Hot Start II High-Fidelity PCR Master Mix (Thermo Scientific, USA) could amplify this region, albeit with some minor non-specific bands, and was therefore selected for use in subsequent experiments. Amplification of the 6.8 kb product increased linearly over a comparable range of cycles to those previously reported^5^ (Supplementary Fig. 2), and as such the number of PCR cycles was maintained as per that work. Optimisation of primer concentrations, annealing temperatures, extension times, and template amounts failed to eliminate the non-specific PCR products.

Since non-specific amplification of the target is likely to compromise the accuracy of the assay we selected an alternative 1.6 kb target region of *TP53*, located further away from the predicted G-quadruplex loci, for investigation. This region is selectively targeted by nitrogen mustard alkylating agents^15^. Additionally, this shorter amplicon allowed the use of standard *Taq* polymerase, which is less likely to be impacted by complex DNA templates^14^. Amplification was linear from 31–40 PCR cycles (Supplementary Fig. 2), and over 10–150 ng template DNA. On the basis of these data, subsequent experiments were performed using 50 ng input DNA and 35 PCR amplification cycles.

**Figure 2:**
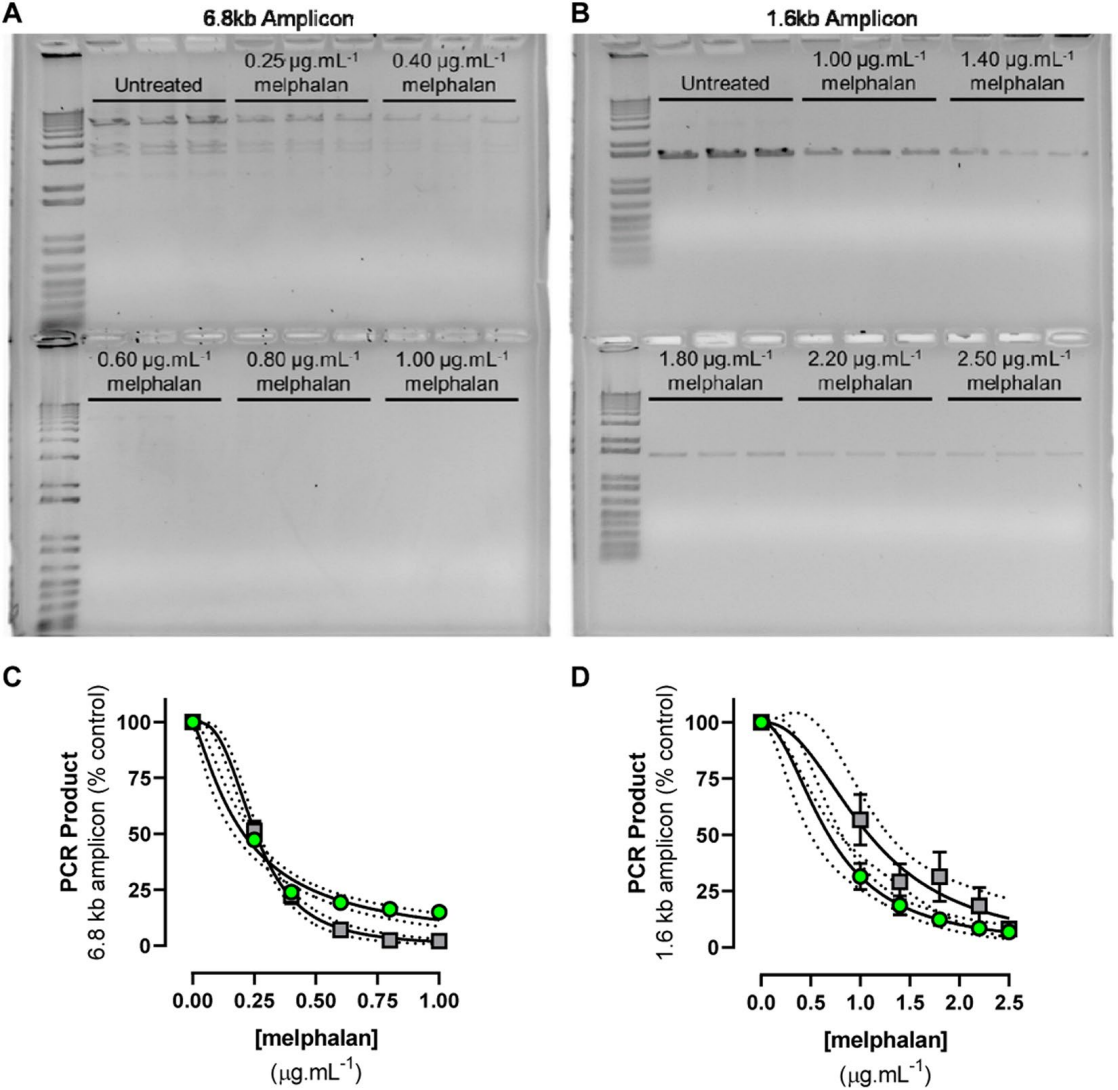
Detection of QPCR-block following exposure of naked gDNA to increasing concentrations of melphalan. The 6.8 kb and 1.6 kb *TP53* amplicon PCR products, analysed in triplicate, were separated by gel electrophoresis alongside size markers and visualised by ethidium bromide staining (**A**,**B**). The gels were then analysed by densitometry (**C**,**D**; grey squares). Parallel analysis of each PCR product was then undertaken using fluorescence spectrophotometry (**C**,**D**; green circles). Data are shown as the mean ± standard error across three independent experiments. Curve fits and IC_50_ values (mean ± standard error) were: 6.8 kb gel densitometry, r^2^ = 0.99, IC_50_ = 0.256 ± 0.007 µg.mL^−1^ and 1.6 kb gel densitometry, r^2^ = 0.86, IC_50_ = 1.088 ± 0.111 µg.mL^−1^. 6.8 kb fluorescence spectrophotometry, r^2^ = 0.98, IC_50_ = 0.207 ± 0.017 µg.mL^−1^, and 1.6 kb fluorescence spectrophotometry, r^2^ = 0.98, IC_50_ = 0.680 ± 0.076 µg.mL^−1^. There was no significant difference in the IC_50_ values obtained by gel densitometry and fluorescence spectrophotometry for either amplicon. Gel images were obtained with an exposure between 0.6–2.7 seconds using Gel Doc^TM^ EZ Imager (Bio-Rad, USA).

The ability of the modified semi-long-range QPCR-block assay to detect melphalan-induced adducts in purified naked gDNA was then assessed and compared with long-range amplification of the 6.8 kb template. Exposure of naked gDNA to increasing concentrations of melphalan resulted in concentration-dependent inhibition of PCR amplification. Differences in the extent of QPCR-block could be reliably detected by gel-densitometry between 0.25 and 1.0 µg.mL^−1^ melphalan for the 6.8 kb amplicon, and from 1.0 to 2.5 µg.mL^−1^ melphalan for the 1.6 kb amplicon (example gels shown in Fig. 2A,B). The resulting concentration-response curves gave IC_50_ values for PCR inhibition of 0.256 ± 0.007 µg.mL^−1^ and 1.088 ± 0.111 µg.mL^−1^ melphalan for the 6.8 and 1.6 kb amplicons, respectively (Fig. 2C,D, grey squares). This 4.25-fold difference in sensitivity directly correlated with amplicon size.

The previous assay^5^ used a short amplicon (0.5 kb, *IFNB1*) as an internal control in a multiplex PCR. However, when we attempted this multiplex approach we observed, by gel densitometry, a significant (*p* = 0.028) decrease in PCR efficiency for formation of the 6.8 kb *TP53* product, but not the *IFNB1* product (Supplementary Fig. 3). Recent QPCR-block protocols for other applications do not include this normalisation step and rely instead on accurate quantification of input gDNA^16^. Importantly we could also detect melphalan-induced inhibition of PCR of this 0.5 kb *IFNB1* sequence using both long-range (IC_50_ = 6.361 ± 1.392 µg.mL^−1^ melphalan) and semi-long-range (IC_50_ = 2.907 ± 2.523 µg.mL^−1^ melphalan) PCR conditions (Supplementary Fig. 4). This suggests that multiplex PCR is not a robust method for normalising the amount of input DNA and differences in PCR reaction efficiency relative to untreated control, and we therefore excluded this approach from further use for this assay.

To improve both the accuracy of gDNA input and importantly the precision of PCR product quantification, we then incorporated fluorescence-based spectrophotometry into our method using the double stranded DNA (dsDNA) intercalating fluorochrome PicoGreen^17^. Lambda dsDNA standards (30–1000 ng.mL^−1^ dsDNA) were used to generate standard curves, with correlation coefficients (r^2^) greater than 0.99 for all standard curves performed. The accuracy of fluorescence-based quantification determined across n = 11 independent standard curves was within 10% for all concentrations tested (Supplementary Fig. 5). The precision of each measurement was high and the coefficients of variation (C_V_) for each sample (n = 3 technical replicates) ranged from 0.07–3.19% (median = 0.66%) for all concentrations tested across those 11 independent standard curves.

The PCR products from the concentration-response curve described previously were then reanalysed using fluorescence spectrophotometry (Fig. 2C,D, green circles). This gave a comparable curve fit to that obtained by gel densitometry for the 6.8 kb amplicon (r^2^ = 0.98 and r^2^ = 0.99, respectively), and a substantially improved curve fit for the 1.6 kb amplicon (gel densitometry: r^2^ = 0.86 and fluorescence spectrophotometry: r^2^ = 0.98). No significant differences in the calculated IC_50_ values were observed between the detection methods (two-sided paired t-test; *p* > 0.05) for either amplicon.

**Figure 3:**
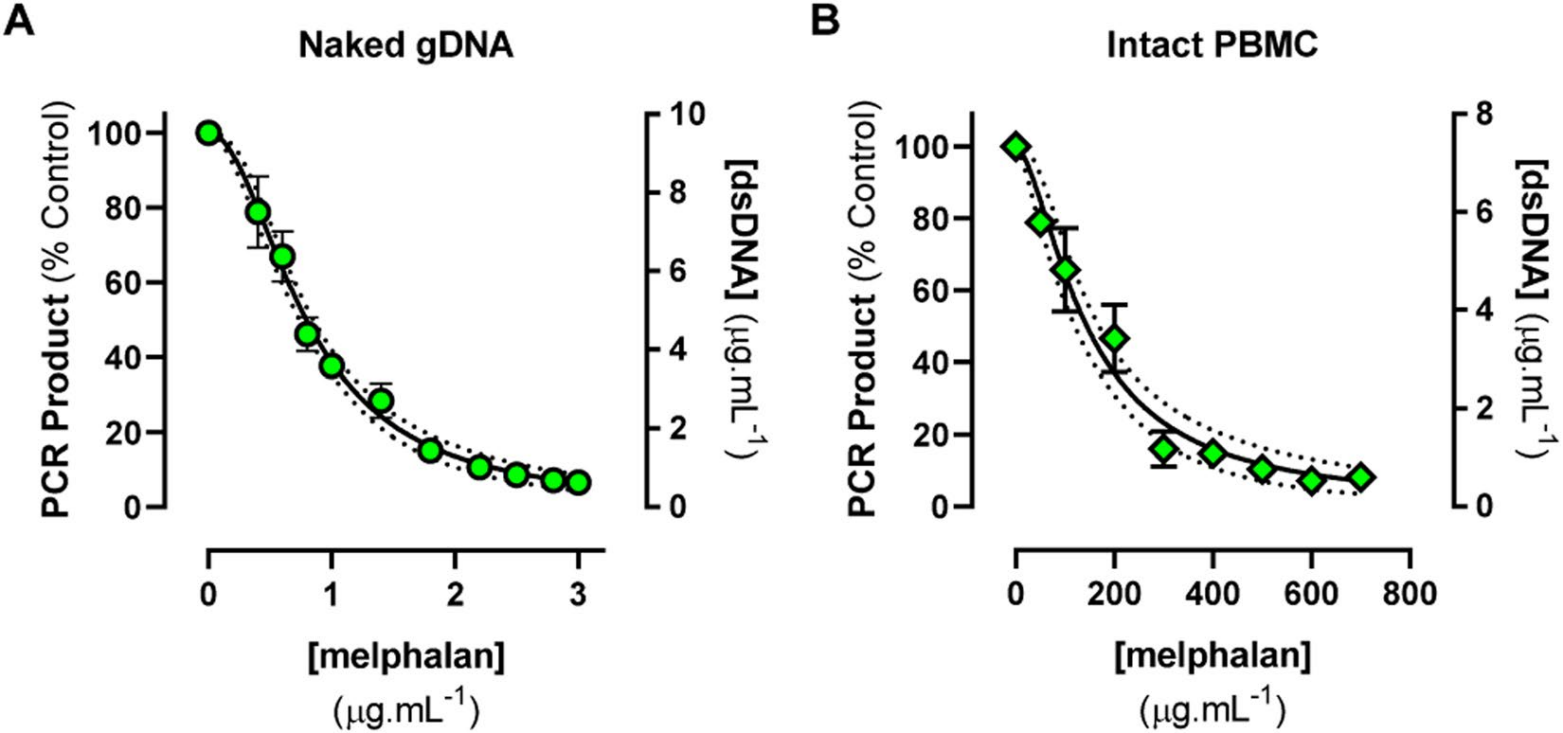
Detection of QPCR-block following exposure of (**A**) naked gDNA (7 µg) and (**B**) intact PBMC (1 × 10^6^ cells) to increasing concentrations of melphalan for 1 h at 37 °C. The 1.6 kb *TP53* amplicon PCR products, analysed in triplicate, were quantified using fluorescence spectrophotometry. Data are shown as the mean ± standard error across three independent experiments. Error bars may be smaller than the symbols. Data are shown relative to untreated control (Y1 axis), and also as directly quantified amplicon (Y2 axis). IC_50_ curve fits with 95% CI (dotted lines) are shown.

To then assess the precision of quantification by fluorescence compared with gel densitometry, two quality control samples (1.4 and 2.2 µg.mL^−1^ melphalan-exposed naked gDNA) were prepared. Intraday precision (Supplementary Fig. 6) across n = 6 PCR replicates of these samples for the 1.6 kb amplicon was unacceptably high when analysed by gel densitometry (C_V_ = 22.9% and 38.8%, respectively). However, assessment of these identical samples using fluorescence spectrophotometry resulted in good intraday precision (C_V_ = 12.19% and 12.35%; n = 6 PCR replicates, each analysed using n = 4 spectrophotometric replicates). The increased precision and sensitivity of the spectrophotometric approach over gel densitometry allowed for an extended range of melphalan concentrations to be tested using the 1.6 kb amplicon (0.4–3.0 µg.mL^−1^ melphalan; Fig. 3A). This resulted in a concentration-response curve with a broader dynamic range than that of the original gel densitometry assay. Additionally, the plate-based spectrophotometry method is suited to higher throughput (e.g. 75 samples per plate versus 18 samples per gel). Furthermore, the plate-based spectrophotometry method allows absolute quantification of the PCR amplicon (µg.mL^_1^ dsDNA), whereas gel densitometry is a semi-quantitative technique that only allows comparative analyses to be undertaken.

To have general clinical utility a straightforward and robust method for analysis of melphalan-induced DNA adducts directly from intact cells is required. To minimise preparation of cells we have implemented a protocol that uses BD Vacutainer® cell preparation tubes to directly collect PBMC from blood samples by centrifugation. Cell viability was ≥99.8% for all PBMC collections (median 100.0%), with a median cell count of 14.2 × 10^6^ viable PBMC obtained per blood draw (range 10.8–17.6 × 10^6^ viable PBMC). Following incubation of 1 × 10^6^ PBMC with melphalan, the cells were treated with a commercially available proteinase K digestion kit, which produces a cell lysate suitable for direct input into PCR without additional DNA purification. Using this approach poor amplification of the 6.8 kb target by Phusion polymerase was observed, likely due to inhibition of this long-range polymerase by human blood cell components. However, *Taq* polymerase amplification of the 1.6 kb target was compatible with the digested PBMC lysate. Formation of the 1.6 kb *TP53* PCR product increased linearly across the full range of lysate volumes tested (1.0–5.0 µL PBMC lysate); all subsequent PCR reactions were undertaken using a fixed volume of PBMC lysate (1.5 µL).

**Figure 4:**
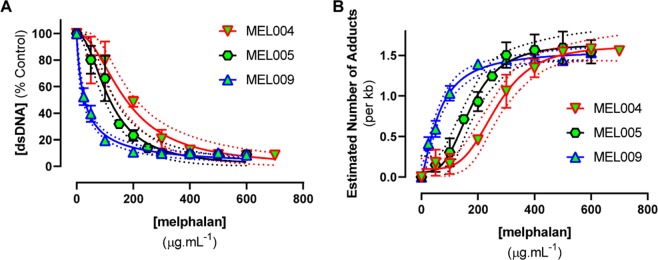
Reproducible detection of melphalan-induced QPCR-block from PBMC collected on three independent occasions from each of three individual PBMC donors (MEL004, MEL005, MEL009). PCR products were quantified using fluorescence spectrophotometry. (**A**) IC_50_ curve fits with 95% CI (dotted lines). (**B**) Estimation of the number of melphalan-induced DNA adducts per kilobase, based on a Poisson equation. Data are mean ± SD of three experimental repeats, each experiment had n = 4 technical repeats at each concentration tested.

Initial determination of QPCR-block following exposure of intact PBMC to melphalan was then undertaken using cells collected from a single donor (MEL003). The range of melphalan concentrations required to achieve QPCR-block were substantially higher for incubation of intact PBMC (Fig. 3B; 50–700 µg.mL-1 melphalan) than for incubation of naked gDNA as described above (Fig. 3A; 0.4–3.0 µg.mL-1 melphalan). This resulted in a 180-fold difference in the IC_50_ for QPCR-block between the naked gDNA (IC_50_ = 0.7933 ± 0.02679 µg.mL^−1^ melphalan) and the intact PBMC from MEL003 (IC_50_ = 144.2 ± 11.86 µg.mL^−1^ melphalan). The biological reproducibility of the intact PBMC assay across data from three independent donations was good (Fig. 3B). Further concentration-response curves were then undertaken using intact PBMC collected from other donors (MEL004, MEL005, MEL009) to further confirm the experimental reproducibility of the IC_50_ data (Fig. 4A). Testing of the effect of melphalan-induced QPCR-block in intact PBMC collected from each individual on three independent donation occasions gave reproducible IC_50_ curves for each donor. We then assessed melphalan-induced QPCR-block in an additional five donors. There was a 7.8-fold difference in IC_50_ observed between the most sensitive (MEL009) and least sensitive (MEL001) donors (Table 1).

**Table 1:** The range of IC_50_ values for melphalan-induced QPCR-block observed across nine individual PBMC donors. All experiments used n = 4 technical replicates for each melphalan concentration tested. N = number of experimental repeats in each donor.

Donor ID	IC_50_ ± SE (µg.mL^−1^ melphalan)	Hill Slope ± SE	r^2^	N
MEL009	27.8 ± 3.0	−0.912 ± 0.080	0.969	3
MEL013	34.7 ± 2.0	−1.226 ± 0.069	0.999	1
MEL008	59.3 ± 1.4	−2.369 ± 0.110	0.998	1
MEL006	72.1 ± 2.7	−1.861 ± 0.108	0.996	1
MEL007	72.4 ± 11.3	−1.302 ± 0.228	0.959	1
MEL005	117.0 ± 9.2	−2.064 ± 0.288	0.904	3
MEL003	144.2 ± 11.9	−1.627 ± 0.182	0.929	3
MEL004	174.3 ± 17.1	−2.039 ± 0.328	0.882	3
MEL001	217.6 ± 12.7	−2.099 ± 0.221	0.985	1

In the published assay^5^, the extent of melphalan-induced inhibition of PCR relative to untreated control was transformed into an estimated amount of DNA damage (number of adducts formed) per 10^6^ nucleotides. This was based on an assumption of random alkylation of DNA sequence by melphalan and the size of the target sequence. Using the same Poisson based transformation we observed a non-linear relationship between the predicted number of adducts and melphalan concentration (Fig. 4B). This is in contrast to the assumption of a simple linear response reported by Stefanou *et al*.^5^, which was based on exposure of a single cell line to a similar range of melphalan concentrations (0–600 µg.mL^−1^).

## Discussion

A logistically simple and cheap method to pre-screen patients for differences in melphalan-induced DNA adduct formation and repair is important since previous studies, using either laborious southern blotting techniques or gel-based QPCR-block, indicate that extent of formation and efficiency of repair of melphalan adducts in *TP53* may influence chemotherapy failure^5,18^. The modified fluorescence spectrophotometry assay described in this report represents a higher throughput, more robust, and more cost effective alternative to the previously published long-range QPCR-block assay using gel-densitometry^5^. Higher concentrations of melphalan were required to cause QPCR-block following exposure of intact PBMC compared with naked gDNA, this reflects the additional impact of other cellular processes including (but not limited to) the amount of melphalan transported into cells, alkylation of other cellular nucleophiles by the drug and the activity of DNA repair enzymes. Importantly, the melphalan concentrations required to achieve QPCR-block following exposure of intact PBMC were identical to that reported previously^5^ using the 6.8 kb sequence and gel-densitometry. It should be noted that these incubation concentrations are above the maximal plasma concentrations of melphalan reported after therapeutic doses (5–15 µg.mL^−1^)^19^. This suggests that whilst QPCR-block is a robust approach for *ex vivo* screening of melphalan-induced DNA damage in primary PBMCs, it is unlikely to be a suitable bioassay for direct therapeutic monitoring.

The QPCR-block approach can be used to detect bulky DNA lesions in many different contexts. For example it can detect the effect of other DNA alkylating agents, such as cisplatin^5^, and the effect of ROS-mediated damage to mitochondrial DNA^20^. An advantage of QPCR compared with global genome wide-DNA damage assays, such as COMET, is that specific gene locations and sequences can be assessed. However, because each application uses different gene sequences, as with any PCR assay careful determination and validation of linear PCR conditions is required.

Importantly we have demonstrated the biological reproducibility of assessment of sensitivity to melphalan-induced QPCR-block using PBMC donated on three separate occasions. We have also detected a 7.8-fold difference in sensitivity to melphalan-induced QPCR-block in nine healthy donors. Inter-individual differences, using gel based long-range QPCR-block, were previously reported for eight healthy volunteers, however this was based on a limited assessment of the effect of only three melphalan concentrations^5^. Due to the higher throughput of our plate based spectrophotometry assay we are able to complete full IC_50_ curves for each donor. This is expected to increase the accuracy of the data for assessment of differences between individuals.

In conclusion, our improved fluorescence-based QPCR-block assay is robust, has good precision, and improved throughput. It also incorporates direct PCR amplification from melphalan exposed-PBMC using commercially available blood tubes and extraction kits to maximise the utility of this assay for future clinical studies.

## Methods

### Study participants

Ethics approval was obtained from the New Zealand Northern A Health and Disability Ethics Committee (16/NTA/239, 18/NTB/170) and all methods were performed in accordance with the relevant guidelines and regulations. Following written informed consent, whole blood was collected from thirteen volunteers. Participants were ≥18 years old, in good health, and on no medication at time of sample collection.

### Genomic DNA extraction

gDNA was extracted from whole blood (8.5 mL) collected in PAXgene Blood DNA Tubes (Qiagen, Germany) using PAXgene Blood DNA Kit (Qiagen, Germany). A NanoDrop 2000 UV-Vis spectrophotometer (Thermo Fisher Scientific Inc., USA) was used to assess gDNA concentration and quality. gDNA was pooled, dispensed into aliquots and stored (−20 °C).

### PBMC collection

Blood samples (8 mL) were drawn into BD Vacutainer® CPT^™^ Tubes (REF 362782, Becton Dickinson and Company, USA). The buffy coat layer was collected by centrifugation (1800 × g, 30 min), transferred into a fresh tube and pelleted (400 × g, 40 min). The supernatant was discarded and PBMC resuspended in phosphate buffered saline (PBS) to a target concentration of 40 × 10^6^ cells.mL^−1^. Cell counts and viability were assessed by trypan blue exclusion on a Neubauer haemocytometer.

### Melphalan incubations

Melphalan (Sigma-Aldrich, USA) was dissolved in 0.5% acidified ethanol (5 mg.mL^−1^) and stored at −20 °C. Aliquots were diluted in either 10 mM Tris-HCl buffer (pH 8.5) or PBS (pH 7.4) for incubation with gDNA or PBMC, respectively. Pooled gDNA (7 µg) or PBMC (1 × 10^6^ cells), were exposed to melphalan (0–2.5 µg.mL^−1^ and 0–700 µg.mL^−1^, respectively) in a final volume of 200 µL, for 1 h at 37 °C. gDNA samples were stored at −20 °C. PBMC were pelleted (400 x g, 40 min), and stored at −20 °C prior to gDNA preparation using the Arturus® PicoPure® DNA Extraction Kit (Applied Biosystems, USA).

### PCR conditions

#### Primer sequences

6.8 kb *TP53* amplicon: forward 5′-TGAGGACCTGGTCCTCTGAC-3′, reverse 5′-TGACGCACACCTATTGCAAG-3′^5^; 1.6 kb *TP53* amplicon: forward 5′-TTCCTCTTCCTACAGTACTCC-3′, reverse 5′-CCTGCTTGCTTACCTCGCT-3′^21^; 0.5 kb *IFNB1* amplicon: forward 5′- ATGAGCTACAACTTGCTTGGA-3′, reverse 5′- TCAGTTTCGGAGGTAACCTGT-3′^5^.

#### Long-range PCR conditions

PCR reactions (50 µL) contained 25 µL Phusion Hot Start II High-Fidelity PCR Master Mix (Thermo Scientific, USA), 0.2 µM of each primer (Invitrogen, Thermo Scientific, NZ), 0.05 µg template gDNA, and an appropriate volume of PCR-grade H_2_O. Optimised PCR cycling conditions: initial denaturation at 98 °C for 30 s; 30 cycles of 98 °C for 10 s, 62 °C for 30 s, 72 °C for 3 min 24 s; and final extension at 72 °C for 10 min.

#### Semi-long-range PCR conditions

PCR reactions (50 µL) contained 25 µL *Taq* PCR Master Mix (Qiagen, Germany), 0.2 µM of each primer (Invitrogen, Thermo Scientific, NZ), either 0.05 µg template gDNA or 1.5 µL PBMC lysate, and an appropriate volume of PCR-grade H_2_O. Optimised PCR cycling conditions: initial denaturation at 94 °C for 3 min; 35 cycles at 94 °C for 30 s, 53 °C for 30 s, 72 °C for 1 min; and final extension at 72 °C for 10 min.

### PCR product quantification

#### Gel electrophoresis and densitometry analysis

The 6.8 and 1.6 kb products were analysed on 0.8% and 1% agarose gels, respectively, alongside a 1 kb Plus DNA ladder (Invitrogen, USA). Electrophoresis was carried out in 0.5X Tris-borate EDTA buffer at 65 V for 160 min (0.8% gel) or at 100 V for 100 min (1% gel). Gels were stained with ethidium bromide (0.05 µg.mL^−1^, 15 min), washed in MilliQ H_2_O (20 min), and then visualised by UV on a Gel Doc™ EZ Imager (Bio-Rad, Hercules, CA, USA). Gel densitometry was undertaken using ImageJ 1.50i software (National Health Institute, MA, USA), with the pixel density of each amplicon band recorded in ‘area density units’.

#### dsDNA quantification by fluorescence spectrophotometry

The Quant-iT PicoGreen dsDNA Assay Kit (Invitrogen, USA) was utilised for this work. In brief, a 1:10 dilution of PCR product (20 µL) was added each well of a 96-well plate, along with 80 µL of 1X TE buffer and 100 µL of PicoGreen solution. This was left to incubate at room temperature protected from light for 5 minutes, after which the fluorescence was measured at 485/20 nm excitation and 528/20 nm emission wavelengths using a BioTeK Synergy 2 microplate reader. Average background fluorescence (100 µL of TE buffer and 100 µL PicoGreen) was subtracted from the data, and the concentration of dsDNA in each well was calculated from a Lambda DNA standard curve (30–1000 ng.mL^−1^; n = 3 technical replicates).

### Data analysis

The estimated number of melphalan-induced DNA adducts was calculated as described in Equation 1 based on the assumption, as previously reported^5^, that adduct formation was randomly distributed. Regression analyses and t-tests were undertaken using GraphPad Prism 8 (GraphPad Prism Software, Inc., CA, USA). Data are presented as the mean ± SE or IC_50_ ± SE across independently repeated experiments, unless otherwise specified. AIC for curve fit comparisons.

Equation 1. Poisson equation to estimate DNA adducts^5^, where N_adduct_ = estimated number of adducts formed per kb, DNA_C_ = amount of dsDNA detected in the control (untreated) PCR reaction, DNA_T_ = amount of dsDNA detected in the melphalan treated (damaged) PCR reaction, A = amplicon size in kb

$${N}_{adduct}=\frac{-ln(\frac{DN{A}_{T}}{DN{A}_{C}})}{A}$$

## Supplementary information

Supplementary information

## Data Availability

Raw data from figures can be provided by corresponding author upon request.
