# Supplementary material for: A higher throughput assay for quantification of melphalan-induced DNA damage in peripheral blood mononuclear cells
Source: Sci Rep. 2019 Dec 11;9:18912. doi: 10.1038/s41598-019-55161-3 (PMC6906414; doi:10.1038/s41598-019-55161-3)
Supplement: Supplementary file 1 — Supplementary information [file 41598_2019_55161_MOESM1_ESM.docx]

A higher throughput assay for quantification of melphalan-induced DNA damage in peripheral blood mononuclear cells

Maia van Kan^1^

Kathryn E Burns^1^

Peter Browett^1^

Nuala A Helsby^1^

^1^Department of Molecular Medicine and Pathology, University of Auckland, Auckland, New Zealand.

Supplementary Figure 1: Four long range polymerases were tested, none of which produced a single PCR product for the 6.8 kb target sequence. The Phusion Hot Start II High-Fidelity PCR Master Mix (Thermo Scientific, USA) gave a major product of the expected size (interpolated size = 6.86 kb, 95% CI 6.25-7.67 kb). The major amplicon for the SequalPrep Long PCR Kit (Life Technologies, USA) was larger than the target sequence (interpolated size = 7.38 kb, 95% CI 6.53-8.62 kb). LA *Taq* DNA Polymerase (TaKaRa, Japan) produced a major amplicon smaller than the target at 6.43 kb (95% CI 5.88-7.14 kb). No PCR products were detected for the Ex Taq Hot Start Version Kit, (TaKaRa, Japan). Small cross symbols (red) indicate the minor products and the large cross symbols (red) indicates the major amplicon produced. The full-length gel electrophoresis images provided for Phusion, SequalPrep, Takara LA and Takara EX are from independent gels. Gel images were obtained with an exposure between 0.6 – 2.7 seconds using Gel Doc^TM^ EZ Imager (Bio-Rad, USA).

Supplementary Figure 2: The range of PCR cycles which gave suitable amplification of 6.8 kb sequence using Phusion Hot Start II High-Fidelity PCR Master Mix and the 1.6 kb sequence using Taq polymerase master mix.

Supplementary Figure 3: The effect of multiplex PCR (inclusion of 0.5kb *IFNB*-1 amplicon) on the efficiency of 6.8 kb amplification.

Supplementary Figure 4: The ability of melphalan to inhibit the amplification of 0.5 kb *IFNB-1* as well as 1.6kb *TP53* sequence. Red cross symbols indicate the size of the amplicon produced.

Supplementary Figure 5: Accuracy and reproducibility of the flurorochrome (picogreen) standard curves used for quantification of PCR amplicon products.

Supplementary Figure 6: Two quality control samples of naked gDNA exposed to 1.4 and 2.2 µg.mL^-1^ melphalan were prepared. N=6 PCR replicates of the 1.6 kb amplicon were then analysed by gel densitometry or quantified by fluorescence spectrophotometry. Intra-day precision was poor when analysed by gel-densitometry (C_V_ 22.9% and 38.8%), but was good when analysed by fluorescence spectrophotometry (C_V_ 12.12% and 12.35%).
